# Medicine

**Published:** 1908-12

**Authors:** J. M. Fortescue-Brickdale


### Progress of the Medical Sciences.

### MEDICINE.

**Arterio-Sclerosis.**—In opening a discussion on this subject at the French Congress of Medicine, Huchard[1] divides the course of the clinical symptoms into four periods, and lays down corresponding rules for treatment. First comes the *pre-sclerotic* or *arterial period*, in which the symptoms are mainly those of an intoxication, its consequent hypertension, and certain painful crises. Renal insufficiency is early and constant, and occurs with or without albuminuria; it tends to increase the toxic symptoms. Asthma or emphysema may also be present, and intensify the condition. The toxic symptoms at this stage are to be treated by a diet mainly composed of milk and vegetables, foods rich in nucleins (sweetbreads, brains, liver, beans and lentils) being absolutely forbidden. Theobromine may be given to increase elimination, with $\frac{1}{6}$ to $\frac{1}{4}$ of a grain of thyminic acid, which increases the solubility of uric acid. The hypertension may be decreased by massage, gymnastic exercises

[1] Huchard, *Gaz. d. Hôp.*, 1908, lxxxi. 1191.

and hydrotherapy: carbo-gaseous baths and diuretic mineral waters are useful. Trinitrin or some similar drug may be prescribed, but the iodides are to be avoided, as they are useless at this stage. Neither high-frequency currents nor the administration of so-called anti-sclerotic sera are of the least value.

The second period he terms the *cardio-arterial*, characterised by manifest lesions in the heart, blood-vessels, and kidneys. The toxic symptoms are more intense, and to these are added insomnia, dyspnœa, tachycardia, arhythmia, and a tendency to dilatation of the heart. Œdema, either visceral or peripheral, may also make its appearance at this stage. The treatment resolves itself into milk diet, with or without some vegetable food, the reduction of chloride intake, together with cardiac tonics, vaso-dilators and diuretics. Very small doses of sodium or potassium iodide (3 to 7 grains) may be given for ten to fifteen days in each month.

The third, or *mitro-arterial period*, is merely an exaggeration of the second. In this all the cardiac cavities and orifices are dilated, there is general dropsy, considerable dyspnœa, and attacks of syncope and enfeebled cardiac action. The amount of urine is much diminished, and albumen is frequently present. Among the complications of this stage are pulmonary œdema and infarction with hæmoptysis, right-sided pleural effusion, and uræmia. The treatment consists of milk diet, digitalin and theobromine.

The fourth period is not reached by all patients, and is marked by the train of symptoms consequent on extreme *cardiac dilatation*. The œdema is extreme and intractable and extends to the serous cavities, the lungs are engorged, and the liver congested and tender to percussion. Drugs are useless; neither digitalis nor theobromine have the least effect. The only measures which will relieve the patient are those which promote diuresis by mechanical means, of which the most potent is the reduction of the intake of fluids. When the urinary flow has been re-established, the ordinary drug treatment may be resumed.

Lancereaux[1], on the other hand, gives larger doses of iodides even in the early stages, especially to the gouty. The iodides should be omitted for one week in each month, and efficient purgatives combined with them to ensure proper elimination. Brine baths and tepid douches are also useful. In France, Rogæ, Brides and Salins-de-Moutiers are recommended for the pre-sclerotic stage; Evian or Vittel when cardiac changes have become established,

In the *symptomatology*, Rudolf[2] calls attention to the cases which occur without marked rise of blood pressure; indeed, occasionally the pressure is actually below normal. This may

[1] Lancereaux, *Bull. Acad. de Méd., Par.*, 1908, 3 s. lix. 597.
[2] Rudolf, *Am. J. M. Sc.*, 1908, cxxxvi. 374.

be due to two causes: the area affected by the sclerosis may be too limited to make any impression on the general arterial pressure, or the heart may have failed. He also points out that many cases do better when the blood pressure is not too much reduced by treatment.

The *cerebral symptoms* have been grouped by Albrecht[1] into two classes: those seen in the slighter cases, and those which characterise advanced or small lesions. Among the former are dizziness, an irritable and emotional temperament, forgetfulness, mental hebetude, and marked susceptibility to the effects of fatigue, alcohol and nicotine.

In the severe forms mental confusion, restlessness and alienation are observed. With these may be combined disturbances of speech without actual stammering, transient localised paresis, increased reflexes and rheumatoid pains in legs and loins. The former type of case is difficult to diagnose from neurasthenia, and the latter from general paralysis; some help may be obtained by noting the condition of retinal arteries.

The *ætiology* of arterio-sclerosis is, as J. Oliver[2] has pointed out, often multiple, though one factor may predominate in any given case. Heredity is an important predisposing cause, but the immediate causes are hyperpiesis, or intoxication of an exogenous or endogenous nature.

Lancereaux considers gout and lead poisoning to be the main precursors of arterio-sclerosis; the influence of tobacco, alcohol and intestinal toxins he thinks has been over-rated.

Huchard[3] has analysed 835 cases out of 15,000 seen in his practice. The histories were as follows:—

Gout, 323; rheumatism, 232; syphilis, 209; abuse of tobacco, 181; acute infectious fevers, 67; diabetes, 48; abuse of alcohol, 27; malaria, 14; climacteric disturbances, 12; nervous and mental disorders, 19.

In 555 no special antecedent conditions could be traced.

Theyer[4], investigating the influence of acute specific fevers, found that among 189 people between the ages of ten and fifty years who had had typhoid, 48 % had palpable arteries, whereas among ordinary healthy persons the percentage was only 17.5. Schlayer[5] found adrenalin-like bodies in normal blood serum, but, curiously enough, persons suffering from chronic nephritis had sera less active in raising blood pressure than normal individuals.

*Recent experimental work.*—A considerable amount of experimental work has been done during the past five years, mainly on rabbits, in which certain arterial lesions may be produced by various methods. José, Erb, Ziegler, and Lissauer have obtained

[1] Albrecht, *Vreljschr. f. gerichtl. Med.*, 1907, 3 F., xxxiii. 83.
[2] G. Oliver, *Clin. J.*, 1908, xxxii. 358.
[3] Huchard, *Bull. Acad. de Méd., Par.*, 1908, 3 s. lx. 59.
[4] Theyer q. F. F. Smith.
[5] Schlayer, *Deutsche Med. Wchnschr.*, 1907, xxxiii. No. 46.

corresponding results, which may be held, therefore, as well authenticated. Adler[1] has summed up this work in a recent paper, from which the following conclusions may be drawn. In rabbits a degenerative process, confined practically to the aorta, and thus *not* resembling the condition known as arterio-sclerosis in man, may be induced experimentally by the injection of various substances. Among these are (1) substances producing a rise of arterial blood pressure, such as adrenaline, ergotine, digalen, barium chloride, hydrastine, and nicotine; (2) substances which either have no effect on arterial blood pressure, or tend to depress it, such as euphthalmine, paraganglin, a mixture of amyl-nitrite and adrenaline, acids, ferments, normal saline, phlorizin and phloretin, lead carbonate in normal saline solution, other metallic salts, and also putrid organic material; (3) bacterial injections—only a few experiments of this nature have been made, but their importance, if confirmed by subsequent experience, is obviously very great. The bacillus typhi and streptococci have been employed with positive results, the most interesting feature in these experiments being that the type of arterial degeneration produced is said to resemble more closely the one common in man.

Adler points out in criticism of these results that although sudden death from cerebral hemorrhage is common in rabbits undergoing injections of adrenaline (which seems to point to degeneration of the smaller cerebral arteries as the result of increased blood pressure), there is not much in these experiments taken as a whole to support the view that hyperpiesis is a common pathological antecedent of arterio-sclerosis. The results are uncertain even in rabbits, and cannot be extended to other animals such as dogs and monkeys. Dosage does not appreciably influence the results, but older animals, and those who are otherwise diseased or in poor condition, are said to react more readily.

Loeper and Boveri[2] found that an excess of calcium salts administered to rabbits rendered them more susceptible to the action of adrenaline, ergotine and pressure substances of that class.

Some interesting experiments by Klotz[3] seem, however, to show that arterial blood pressure may affect the nutrition of the middle coat of the aorta, much in the same way as do adrenaline injections. He suspended medium-sized rabbits by the hind legs for three minutes every day during periods of 92 to 130 days, a proceeding which he showed tended to raise the blood pressure in the aorta. Five rabbits were employed, and all showed changes in aorta, carotids, subclavians and brachials. In the peripheral vessels, in contra-distinction to the aorta, the primary lesion was found in the tunica intima.

When we come to correlate the experimental findings with the

1 Adler, *Am. J. M. Sc.*, 1908, cxxxvi. 241.
2 Loeper and Boveri, *Riforma. Med.*, 1907, xxiii. 986.
3 Klotz, *Centralbl. f. allg. Path. u. Path. Anat.*, July, 1908.

results of clinical experience, certain broad facts seem to stand out clearly. In the first place it is necessary to draw a clear line between inflammatory processes, such as result from lues (endarteritis proliferans and obliterans) and purely degenerative lesions such as atheroma and arterio-sclerosis. Secondly, we must realise that the former is a senile change in the main, whereas the latter is not uncommon in quite young people, and usually begins in the third decade. Simnitzky found among 138 autopsies on persons under twenty-five that 38, or 27.5 %, showed sclerotic changes. Although the experimental evidence that this can be produced by merely raising the blood pressure is not at present very strong, there is considerable, not to say overwhelming, clinical evidence of the importance of this factor in the human subject. Among 4,000 persons of the so-called "labouring" classes examined, it was found that a very high percentage had thickened radial arteries, while in left-handed persons the left radial is found to be affected in some cases where the right is normal.

Toxic conditions in rabbits tend to produce aortic atheroma, whether the toxins raise the arterial pressure or not, and there is some evidence to show that the lesions met with in young persons after acute specific fevers are also of this type.

As regards the experimental evidence on the question of treatment, it is interesting to note that the action of iodine—which, in the form of iodipin, was thought to counteract the effect of adrenalin—has been found by subsequent researches to have no effect. Oil, however, and in especial sesame oil (which enters into the composition of iodipin), has been shown by Koranyi to counteract the digalen[1].

J. M. Fortescue-Brickdale.

### SURGERY.

[1] Koranyi, *Deutsche Med. Wchnschr.*, 1907, No. 5.

